# Comparing Matching Strategies to Address Selection into Palliative Care among Veterans

**DOI:** 10.1093/geroni/igaf122.2006

**Published:** 2025-12-31

**Authors:** Brystana Kaufman, Sandra Woolson, Paul Dennis, Joshua Thorpe, Susan Hastings, David Bekelman, Courtney Van Houtven

**Affiliations:** Duke University, Durham, North Carolina, United States; Department of Veterans Affairs, Durham, North Carolina, United States; VA Durham Health Care System, Durham, North Carolina, United States; The University of North Carolina at Chapel Hill, Chapel Hill, North Carolina, United States; Durham VA Healthcare System, Durham, North Carolina, United States; Department of Veterans Affairs, Auroroa, Colorado, United States; Duke University, Durham, North Carolina, United States

## Abstract

Selection bias influences observational studies of palliative care because many of the reasons for palliative care referral are not documented in administrative data sources, making it difficult to control for unobserved confounders. In observational studies, matching and weighting methods aim to control for unobserved confounding that is correlated with observed characteristics. This study aimed to quantify residual selection bias due to unobserved factors by comparing 3 strategies (combinations of exact match, Mahalonobis distance, and propensity weighting) applied to multi-payer data (2014-2017) to support causal inference approaches. The national cohort included veterans with life limiting conditions who were new users of VA specialty outpatient PC (treat) and matched veterans with non-PC specialty outpatient encounter (active comparator). Residual unobserved confounding was measured by comparing survival curves between PC and non-PC groups at 90 days. Of 5820 unique new PC users, 1527, 2811, and 4381 pairs were matched in strategy 1, 2, and 3, respectively. Survival at 90 days were 14.1, 15.8, and 17.8 percentage points lower compared to matched veterans in strategies 1,2, and 3 respectively. In subgroup analyses, the smallest difference in survival between groups was among veterans with prior inpatient PC (4.4 percentage points). Matching on observed characteristics was not sufficient to control for selection into PC, as illustrated by differences in survival following an initial PC compared to non-PC specialty encounter among veterans with life limiting conditions. Further work is needed to understand the impact of residual confounding on estimates for the effect of PC on veteran outcomes.

